# Recyclable Choline Nicotinate and Ferulate Aqueous Solutions as Efficient Lignin Solvents

**DOI:** 10.3390/polym10080840

**Published:** 2018-07-30

**Authors:** Airong Xu, Lin Chen, Xingmin Xu, Zhihong Xiao, Rukuan Liu, Ruixue Gao, Mengzhen Yuan, Luwei Zhang

**Affiliations:** 1 School of Chemical Engineering and Pharmaceutics, Henan University of Science and Technology, Luoyang, Henan 471003, China; chenlin_0212@126.com (L.C.); xxm78v@126.com (X.X.); 18438699882@163.com (R.G.); shiyuejianjia@163.com (M.Y.); 15757565627@163.com (L.Z.); 2 Hunan Academy of Forestry, Changsha, Hunan 410004, China; xzhh1015@163.com

**Keywords:** lignin, H_2_O/choline carboxylate solvent, dissolution mechanism, recycling, regenerated lignin properties

## Abstract

Four novel choline carboxylate aqueous solution systems were developed by mixing H_2_O with choline nicotinate [Ch][Na], choline ferulate [Ch][Fa], choline vanillate [Ch][Va] and choline syringate [Ch][Sa]. The solubility of lignin in the four solvents was determined at 25 °C. The influence of the molar ratio of H_2_O to [Ch][Na] ([Ch][Fa], [Ch][Va] and [Ch][Sa]) and the anionic structure on lignin solubility were systematically investigated. It was found that, the anionic structure and H_2_O content significantly affected lignin dissolution. Interestingly, H_2_O/[Ch][Na] and H_2_O/[Ch][Fa] solvents show efficient capacity for lignin dissolution even at room temperatures. The dissolution of lignin in H_2_O/[Ch][Na] and H_2_O/[Ch][Fa] solvents is mainly ascribed to the interaction of lignin with the alkyl chain in the anion and cation dissociated from [Ch][Na]([Ch][Fa]) by H_2_O. In addition, the recycling of the lignin solvent was examined, and the structure and thermostability of the lignin regenerated from the solvent were also estimated.

## 1. Introduction

With the rapid depletion of fossil resources, the production of chemicals and materials from renewable lignocellulosic biomass in place of fossil resources is attracting much attention [1]. Lignin is one of the three principal components (lignin, cellulose and hemicellulose) of lignocellulosic biomass [2,3]. At the same time, lignin is also the second most abundant biopolymer in nature next to cellulose and the only native biopolymer on the Earth that contains aromatic phenolpropanoid monomers. Lignin has been regarded as a promising alternative to fossil resources. Lignin is a cross-linked amorphous copolymer synthesized from random polymerization of three primary phenylpropane monomers, coniferyl alcohol, paracoumaryl alcohol and sinapyl alcohol, which are bonded together through several different C–O–C and C–C interunit linkages [4]. The complex structure results in low solubility or insolubility in classical organic solvents and water [5], which is one of the main challenges for the efficient utilization of lignin. Therefore, many efforts have been made for improving lignin dissolution.

Over the past years, ionic liquids (ILs) have been utilized to dissolve/process lignocellulosic biomass in view of their outstanding properties such as non-detectable vapor pressure, physicochemical tunabilities, recoverability, and so on [6,7,8,9,10,11,12,13,14,15,16]. The reported ILs for dissolving/processing lignin include imidazolium-based ILs [17,18,19], ammonium (phosphonium and pyrrolidinium) based ILs [20], pyridinium carboxylate ILs [21], and bio-derived ILs which are composed of ions derived from naturally occurring bases (e.g., choline) and acids (e.g., amino acids and carboxylic acids) [22,23]. However, the ILs are usually expensive, very viscous or toxic and the bio-derived ILs exhibit poor dissolution capacity for lignin. In this context, some IL–water solutions were developed as lignin solvents. Wang et al. found that some aqueous dialkylimidazolium-based IL solution systems could dissolve lignin at 60 °C, and 39.8 g/100 g of the maximum lignin solubility was obtained in aqueous 1-ethyl-3-methylimidazolium acetate ([C_2_mim][CH_3_COO]) solution containing 70 wt % of [C_2_mim][CH_3_COO] [24]. Recently, binary solvent systems consisting of *γ*-valerolactone + water (dimethyl sulfoxide, *N,N*-dimethylformamide or ILs) have been reported to efficiently dissolve various types of lignin [25]. It has also been reported that two novel solvents including eco-friendly polysorbate aqueous solvents and aqueous glycol solvents display excellent dissolving capacity for lignin [26,27]. Very recently, Xu et al. developed 13 novel kinds of choline carboxylate/H_2_O solvents for lignin [28]. The authors find that the solubility of lignin increases with increasing alkyl chain length in the carboxylate anions, and a substitution of H in carboxylate anions by the OH or NH_2_ group as well as the use of choline di-/tri-carboxylates leads to the decrease of lignin solubility, or even makes the lignin insoluble in the solvents.

Although previous investigations revealed some important aspects in the development of lignin solvents, the dissolution performance of lignin is unknown in H_2_O/[Ch][Na], H_2_O/[Ch][Fa], H_2_O/[Ch][Va] and H_2_O/[Ch][Sa] solvents. Therefore, in this work, [Ch][Na], [Ch][Fa], [Ch][Va] and [Ch][Sa] were synthesized. At the same time, their thermal properties melting/glass transition and thermal decomposition temperatures were also determined for safe application. Then H_2_O/[Ch][Na], H_2_O/[Ch][Fa], H_2_O/[Ch][Va] and H_2_O/[Ch][Sa] solvents were obtained by mixing H_2_O with [Ch][Na], [Ch][Fa], [Ch][Va] and [Ch][Sa]. Furthermore, the solubilities of lignin in the solvents were determined at 25 °C, the effects of the H_2_O/[Ch][Na], H_2_O/[Ch][Fa], H_2_O/[Ch][Va] and H_2_O/[Ch][Sa] molar ratios and the anionic structure on lignin solubility were systematically investigated, and the possible dissolution mechanism was proposed. In addition, the lignin generated from choline carboxylate/H_2_O/lignin solution was characterized by Fourier transform infrared (FT-IR) spectroscopy, thermogravimetric analysis (TGA) and molecular weight examination. The selection of [Ch][Na], [Ch][Fa], [Ch][Va] and [Ch][Sa] is based on the fact that choline cation is biodegradable [29], and the paired anions [Na]^−^, [Fa]^−^, [Va]^−^ and [Sa]^−^ are derived from nicotinic acid, ferulic acid, vanillic acid and syringic acid, respectively. Nicotinic acid is vitamin B3, and ferulic acid, vanillic acid and syringic acid are often used as medicine, spice and food additives.

## 2. Materials and Experiment

### 2.1. Materials

Lignin (alkali) with a moisture content of 5% was purchased from Sigma-Aldrich (St. Louis, MO, USA). The enzymatically hydrolyzed lignin, isolated from cellulolytic enzyme hydrolysis of corncob, was from Shandong Longlive Bio-Technology Co., Ltd. (Shandong, China) [30]. The two lignins were dried before use under vacuum at 60 °C; choline hydroxide aqueous solution (46%, *w*/*w*) was from Alfa Aesar (Haverhill, MA, USA); nicotinic acid (98.0%), ferulic acid (99.0%), vanillic acid (98.0%), and syringic acid (98.0%) were purchased from Aladdin Industrial Corporation (Shanghai, China); deuterated DMSO (DMSO-*d*_6_) used for nuclear magnetic resonance (NMR) spectra examination was purchased from Qingdao Weibo Tenglong Technol. Co. Ltd (Qingdao, China). These materials were used as received. Distilled water was used throughout the experiments.

### 2.2. Synthesis of [Ch][Na], [Ch][Fa], [Ch][Va] and [Ch][Sa]

[Ch][Na], [Ch][Fa], [Ch][Va] and [Ch][Sa] were synthesized by using the procedure described in the literatures [22,23]. Briefly, an aqueous solution of choline hydroxide [Ch][OH] was neutralized with equal molar nicotinic acid to obtain [Ch][Na] aqueous solution. Water in [Ch][Na] aqueous solution was then removed by evaporation under reduced pressure. The [Ch][Na] obtained was finally dried under vacuum. [Ch][Fa], [Ch][Va] and [Ch][Sa] were synthesized by a similar procedure. In addition, measurements of the thermal properties of [Ch][Na], [Ch][Fa], [Ch][Va] and [Ch][Sa] are included in the Supplementary Materials.

### 2.3. Dissolution of Lignin

As a representative, water, at a given molar ratio, was added to [Ch][Na] to obtain [Ch][Na]/H_2_O solvent. This solvent was generally prepared prior to use. Lignin was added to 2.0 g of [Ch][Na]/H_2_O solvent in a 25 mL glass-stoppered colorimetric tube. The tube was then immersed in an oil bath (DF-101S, Gongyi Yingyu Instrument Factory, Gongyi, China). The temperature instability was estimated to be ±0.5 °C. The lignin/[Ch][Na]/H_2_O mixture in the tube was stirred at 25.0 °C. Additional lignin was added after the lignin in the tube was completely solubilized, based on observations using a polarizing microscope. When lignin became saturated to the point that no more lignin was dissolved further, its solubility (expressed by gram per 100 g of solvent) at 25 °C was calculated from the amount of the solvent and lignin added.

### 2.4. Characterization of the Regenerated Lignin

Fourier transform infrared (FT-IR) spectra were determined on a Necolet Nexus spectrometer (Nicolet iN10, Thermo Fisher Scientific, Waltham, MA, USA) to analyze the functional groups in the regenerated lignin samples with KBr pellets. The FT-IR spectra for each sample were collected for a total of 16 scans at a resolution of 2 cm^−1^ within the wavenumber range from 4000 to 400 cm^−1^. Thermogravimetric analysis (TGA) was conducted on a NETZSCH STA 449 C thermal analyser (Netzsch Corporation, Freistaat Bayern, Germany) using alumina crucibles under flowing N_2_ at a heating rate of 10 °C min^−1^. The sample mass for each measurement was ca. 10–15 mg. The number averaged (*M*_n_) and weight averaged molecular weight (*M*_w_) were examined on a Waters e2695 chromatographic instrument (Waters, MA, USA). *N,N*-Dimethylformamide was used as mobile phase. Calibration of lignin molecular weight was achieved using polystyrene calibrants.

### 2.5. Measurements of ^13^C NMR Spectra

Measurements of ^13^C NMR spectra for [Ch][Na] in D_2_O/[Ch][Na] (*R =* 10) solvent and D_2_O/[Ch][Na] (*R =* 10)/lignin (8 wt %) solution were performed at room temperature on a Bruker Avance-400 NMR spectrometer (Bruker Corporation, Rheinstetten, Germany) operating at 400.13 MHz. D_2_O was used as deuterated solvent and co-solvent in place of H_2_O for the convenience of ^13^C NMR measurements due to the similarity of D_2_O with H_2_O. Chemical shifts were given in ppm downfield from TMS.

## 3. Results and Discussion

### 3.1. Thermal Properties of [Ch][Na], [Ch][Fa], [Ch][Va] and [Ch][Sa]

In view of the safe application and no reported thermal property data of [Ch][Na], [Ch][Fa], [Ch][Va] and [Ch][Sa], the thermal properties for them were determined and presented in Table 1. It can be seen that the melting temperatures *T*_m_ or glass transition temperatures *T*_g_ range from −7 °C to 103 °C. This indicates that, for the choline carboxylates with the same [Ch]^+^ cation, *T*_m_/*T*_g_ considerably depends on anionic structure. Moreover, the replacement of the hydrogen atom of the benzene ring in carboxylate anion by methoxy group significantly leads to a decrease in *T*_g_. Consequently, *T*_g_ of [Ch][Sa] (−2 °C) is markedly lower than that of [Ch][Va] (103 °C). At the same time, it was also found that both the increase of alkyl chain length and the addition of ethylenic bond in the carboxylate anion also significantly results in a decrease in *T*_g_. For example, *T*_g_ of [Ch][Fa] (−7 °C) is notably lower than that of [Ch][Va] (103 °C). In addition, *T*_d_ is also influenced by the anionic structure, and is 163 °C for [Ch][Fa], 171 °C for [Ch][Sa], 175 °C for [Ch][Va], and 219 °C for [Ch][Na], respectively (See Table 1).

### 3.2. Effect of Anionic Structure

Table 2 gives the solubility data of lignin in [Ch][Na]/H_2_O, [Ch][Fa]/H_2_O, [Ch][Va]/H_2_O and [Ch][Sa]/H_2_O solvents. For the solvents investigated in this work, lignin solubility is significantly affected by anionic structure. For example, [Ch][Na]/H_2_O and [Ch][Fa]/H_2_O solvents can efficiently dissolve lignin at a proper molar ratio range. However, lignin is not soluble at all in [Ch][Va]/H_2_O and [Ch][Sa]/H_2_O solvents upon replacing the hydrogen atom of the benzene ring in carboxylate anion by methoxy and/or hydroxyl groups. In addition, the solubilities of lignin in [Ch][Na]/H_2_O solvents are generally higher than those in [Ch][Fa]/H_2_O solvents. As a matter fact, the addition of 57 wt % lignin to [Ch][Na]/H_2_O (*R =* 3:1–10:1) gave a highly viscous liquid that was hard to stir, and we could not determine the final solubilities of lignin in the solvents. A similar trend is observed for [Ch][Fa]/H_2_O solvents.

At the same time, as a representative, we also determined the solubility of the enzymatically hydrolyzed lignin which has a similar structure to lignin in untreated lignocellulosic biomass [18], and the solubility data of the lignin are given in Table 3. Apparently, [Ch][Fa]/H_2_O solvents still exhibit powerful dissolution capacity for the enzymatically hydrolyzed lignin.

### 3.3. Effect of H_2_O Addition

At 25 °C, lignin is not soluble in H_2_O or [Ch][Fa], and [Ch][Na] is solid (see Table 2). Interestingly, as H_2_O is added to [Ch][Na] and [Ch][Fa], lignin becomes readily soluble. However, it is also noted that at all molar ratio ranges for [Ch][Va]/H_2_O and [Ch][Sa]/H_2_O solvents, lignin is not soluble at all. This further indicates that the anionic structure of the choline carboxylate in the H_2_O/choline carboxylate solvent dominates the dissolution of lignin. It has been reported that H_2_O can partially dissociate an electrolyte into a free cation and anions [31]. Therefore, after the addition of H_2_O to the choline carboxylate, the choline carboxylate is partially dissociated into free [Ch]^+^ cation and carboxylate anions which interact with lignin and promote the dissolution of lignin. With respect to the insolubility of lignin in [Ch][Va]/H_2_O and [Ch][Sa]/H_2_O solvents, it may be due to the interactions of [Ch][Va]/[Ch][Sa]–H_2_O being stronger than those of the [Ch][Va]/[Ch][Sa]–lignin.

Moreover, it is also found that, in [Ch][Na]/H_2_O and [Ch][Fa]/H_2_O solvents, the lignin solubility increases with increasing H_2_O content followed by reaching the maximum solubility. However, when the H_2_O content further increases, the lignin solubility dramatically decreases. As mentioned above, the choline [Ch]^+^ cation and carboxylate anion in [Ch][Na]/H_2_O and [Ch][Fa]/H_2_O solvents dominate the lignin dissolution. Moreover, the concentrations of the dissociated choline [Ch]^+^ cation and carboxylate anion increase with increasing H_2_O content. Hence, it is easy to understand why the lignin solubility increases with increasing H_2_O content. However, the further increase in H_2_O content decreases the concentration of the dissociated choline [Ch]^+^ cation and carboxylate anion, and thus the lignin solubility decreases with increasing H_2_O.

### 3.4. Interaction between Lignin and [Ch][Na] in H_2_O/[Ch][Na] Solvent

**Figure 1:**
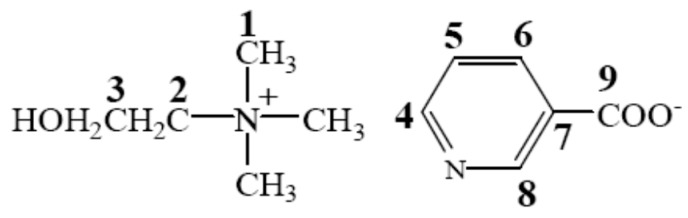
The schematic structure and carbon atom numbering of [Ch][Na].

To investigate the interaction in the H_2_O/[Ch][Na]/lignin solutions, the ^13^C NMR measurements of [Ch][Na] in H_2_O/[Ch][Na] (*R =* 10) solvent and H_2_O/[Ch][Na] (*R =* 10)/lignin (8 wt %) solution were performed. The results are given in the Supplementary Materials (Figures S1 and S2), and the corresponding ^13^C NMR data are given in Table 4. At the same time, Figure 1 gives the schematic structure and the numbering of the C atoms in [Ch][Na] to help understanding.

Based on the ^13^C NMR data in Table 4 that, after lignin was dissolved in H_2_O/[Ch][Na] solvent, the C9 signal in nicotinate anion slightly shifts upfield (chemical shift decreases). This is mainly ascribed to the strong interaction of the carboxyl group in nicotinate anion with H_2_O, which disables the carboxyl group to interact with lignin. At the same time, it is noted that the signals of the carbon atoms C1–C8 except for C7 shift upfield (a decrease in the chemical shifts). This suggests that the aromatic ring or/and alkyl units in cations and anions interact with lignin, resulting in the increase of the electron cloud density of these atoms. Therefore, the dissolution of lignin in H_2_O/[Ch][Na] solvent mainly results from the interaction of the alkyl chain instead of the carboxyl group in nicotinate anions, which is similar to the results reported in the literature [26]. To verify this speculation, the solubility of lignin in aqueous choline chloride (H_2_O/[Ch]Cl) solutions was determined at 25 °C at different molar ratios of H_2_O to [Ch]Cl. [Ch]Cl was chosen because there is no alkyl chain in its anion. It was found that lignin was not dissolved in H_2_O/[Ch]Cl solvents at any molar ratio of H_2_O to [Ch]Cl. This further indicates that the aromatic ring or/and alkyl units in the cations and anions played a key role in lignin dissolution. Additionally, in H_2_O/[Ch][Va]([Ch][Sa]) solvents, the replacement of H of benzene ring in carboxylate anions by OH and OCH_3_ group resulted in the remarkable enhancement of hydrophilicity of the carboxylate anions, which strengthens IL-H_2_O interaction. Therefore, lignin was insoluble in H_2_O/[Ch][Va]([Ch][Sa]) solvents. At the same time, it is also noted that [Fa]^−^ and [Va]^−^ anions have the same benzene ring as well as the methoxy and hydroxyl groups, and lignin is soluble in the H_2_O/[Ch][Fa] solvents at *R* = 4–15 owing to the existence of CH=CH in the [Fa]^−^ anion, but not soluble in the H_2_O/[Ch][Sa] solvents at all molar ratio ranges. This further indicates that the aromatic ring and/or alkyl units in the cations and anions are favorable to lignin dissolution.

### 3.5. Recovery of Solvent and Structure and Thermal Properties of the Regenerated Lignin

Recovery of H_2_O/choline carboxylate solvent was estimated. After the complete dissolution of lignin in the H_2_O/choline carboxylate solvent, lignin can be regenerated and the H_2_O/choline carboxylate solvent can be recovered by adding additional water. In a typical recovery trial, 4.0 g of H_2_O/[Ch][Na] (*R =* 8:1) solvent and 40.0 wt % lignin solution were used. The lignin solution was filtered using a 60 mL sand-core filter funnel to obtain lignin, and the lignin was then washed 4–5 times by 50 g H_2_O to ensure that [Ch][Na] had been washed out. The recovered lignin was about 89 wt %. All filtrate was collected together. The H_2_O/[Ch][Na] solvent recovered could be obtained by evaporating off H_2_O. Interestingly, after three dissolving-recovering cycles, the solvent still displayed the same dissolution capacity of lignin as the original solvent.

The averaged molecular weight is shown in Table 5. The molecular weight of the regenerated lignin from [Ch][Fa]/H_2_O (*R =* 10:1)/lignin solution is close to that of original lignin, indicating that the molecular structure of the regenerated lignin is hardly disrupted. The molecular weight of the regenerated lignin from [Ch][Na] (*R =* 8:1)/H_2_O/lignin solution is slightly higher than that of the original lignin. This is mainly ascribed to the fact that in the regeneration processes, the lignins of some small number weight are washed off.

**Figure 2:**
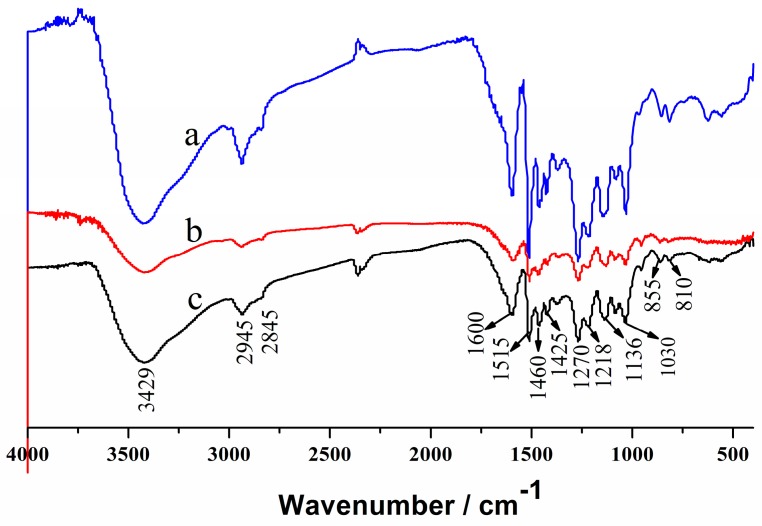
FT-IR spectra of lignin: (**a**) the original lignin; (**b**) the regenerated lignin from [Ch][Fa]/H_2_O (*R =* 10:1)/lignin solution; (**c**) the regenerated lignin from [Ch][Na] (*R =* 8:1)/H_2_O/lignin solution.

Figure 2 shows the FT-IR spectra of the original lignin and the regenerated lignin. The FT-IR spectra for the regenerated lignins from [Ch][Fa]/H_2_O (*R =* 10:1)/lignin solution and [Ch][Na] (*R =* 8:1)/H_2_O/lignin solution are in excellent agreement with those from the original lignin, hence confirming that no chemical reaction occurs between the solvents and lignin in the dissolution and regeneration processes. The detailed affiliation for the absorption bands of the original and regenerated lignin are placed in Supplementary Materials. The FT-IR spectra of the original and regenerated lignin are similar to those reported in the literature [32,33,34].

**Figure 3:**
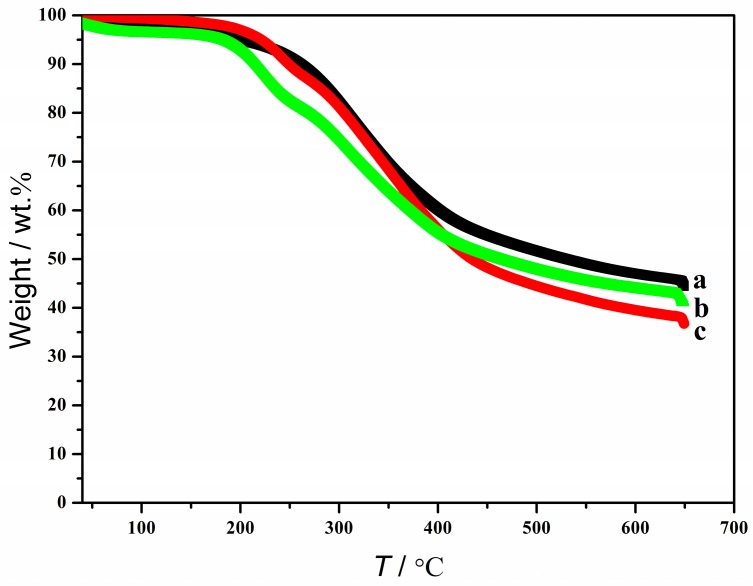
Thermal decomposition profiles of lignin: (**a**) the original lignin; (**b**) the regenerated lignin from [Ch][Fa] (*R =* 10:1)/H_2_O/lignin solution; (**c**) the regenerated lignin from [Ch][Na] (*R =* 8:1)/H_2_O/lignin solution.

Figure 3 shows the TGA curves of the original lignin and regenerated lignin. It can be seen that the regenerated lignins from H_2_O/[Ch][Na] (*R =* 10:1)/lignin solution (244 °C) and H_2_O/[Ch][Fa] (*R =* 10:1)/lignin solution (204 °C) exhibit a slightly lower onset temperature for the decomposition compared to the original lignin (260 °C). It is likely that the lower molecular weight lignins are washed away after the dissolution and regeneration process. Moreover, it is also found that, upon heating to 650 °C, the residual char yield of the original lignin is about 45 wt %, and the residual char yield of the regenerated lignin from H_2_O/[Ch][Na] (*R =* 10:1)/lignin solution is about 38 wt % and 43 wt % for the regenerated lignin fron H_2_O/[Ch][Fa] (*R =* 10:1)/lignin solution.

## 4. Conclusions

Novel solvents have been developed by adding H_2_O to [Ch][Na], [Ch][Fa], [Ch][Va] and [Ch][Sa]. Anionic structure significantly affects the lignin solubility in the choline carboxylate/H_2_O solvents. [Ch][Na]([Ch][Fa])/H_2_O solvents can readily dissolve lignin even at ambient temperatures, but lignin is not insoluble in [Ch][Va]([Ch][Sa])/H_2_O solvents due to the replacement of the hydrogen atom in the benzene ring in the benzoate anion by methoxy and/or hydroxyl groups. The dissolution of lignin in [Ch][Na]([Ch][Fa])/H_2_O solvents mainly results from the interaction of the alkyl chain in [Ch][Na]([Ch][Fa]) with lignin, and the role of H_2_O serves to dissociate [Ch][Na] and [Ch][Fa] into free anions and cations. [Ch][Na]([Ch][Fa])/H_2_O solvents can be recovered and reused, and the recovered solvent still displays the same dissolution capacity of lignin as the original solvent after three dissolving–recovering cycles. Moreover, the regenerated lignin exhibits good thermal stability and hardly disrupted molecular structure according to FT-IR, TGA and molecular weight investigations.

## Figures and Tables

**Table 1:** Melting temperature (*T*_m_), glass transition temperature (*T*_g_), and thermal decomposition temperature (*T*_d_).

IL	Abbreviation	Schematic Stucture		*T*_m_ (°C)	*T*_g_ (°C)	*T*_d_ (°C)
		Cation	Anion			
Choline nicotinate	[Ch][Na]	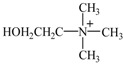	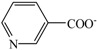	76.9	— ^a^	219.0
Choline ferulate	[Ch][Fa]		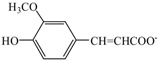	— ^a^	−7.2	163.1
Choline vanillate	[Ch][Va]		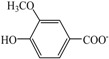	— ^a^	102.9	174.8
Choline syringate	[Ch][Sa]		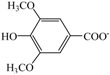	— ^a^	−1.6	170.6

^a^ Not observed.

**Table 2:** Solubility of lignin (alkali) in the H_2_O/choline carboxylate solvent at 25 °C.

*R*	Solubility (g/100 g Solvent)		
	[Ch][Na]	[Ch][Fa]	[Ch][Va] and [Ch][Sa]
0	— ^a^	— ^a^	— ^a^
1	— ^a^	— ^a^	— ^a^
2	0.4	— ^a^	— ^a^
3	>57	— ^a^	— ^a^
4	>72	29	— ^a^
6	— ^b^	>42	— ^a^
7	>72	— ^b^	— ^a^
8	>69	— ^b^	— ^a^
10	>69	>51	— ^a^
15	— ^a^	>50	— ^a^
16	— ^a^	— ^a^	— ^a^
17	— ^a^	— ^a^	— ^a^

*R* is the mole ratio of H_2_O to choline carboxylate. ^a^ Insoluble at the given molar ratio. ^b^ Not measured.

**Table 3:** Solubility of lignin (enzymatically hydrolyzed) in the [Ch][Fa]/H_2_O solvents at 25 °C.

Entry	Solvent	Solubility (Gram Per 100 g of Solvent)
1	[Ch][Fa]/H_2_O (*R* = 4:1)	31.0
2	[Ch][Fa]/H_2_O (*R* = 6:1)	>45.0
3	[Ch][Fa]/H_2_O (*R* = 10:1)	>51.0
4	[Ch][Fa]/H_2_O (*R* = 15:1)	>50.0

*R* is the molar ratio of H_2_O to [Ch][Fa].

**Table 4:** ^13^C nuclear magnetic resonance (NMR) chemical shifts (*δ*) of [Ch][Na] in H_2_O/[Ch][Na] (*R =* 10:1) solvent and H_2_O/[Ch][Na] (*R =* 10:1)/lignin solutions.

Lignin Concentration (wt %)	C1	C2	C3	C4	C5	C6	C7	C8	C9
0	53.43	55.17	67.10	148.83	123.62	132.52	137.19	149.80	171.08
8.0	53.38	55.14	67.06	148.78	123.58	132.49	137.20	149.71	171.05
Δ*δ*	−0.05	−0.03	−0.04	−0.05	−0.04	−0.03	0.01	−0.09	−0.03

**Table 5:** The number averaged (M_n_) and weight averaged molecular weight (M_w_) of samples.

Sample	M_n_	M_w_
Regenerated lignin from [Ch][Fa]/H_2_O (*R =* 10:1)/lignin solution	2,062,903	2,738,281
Regenerated lignin from [Ch][Na] (*R =* 8:1)/H_2_O/lignin solution	2,244,528	2,842,232
Original lignin	2,056,690	2,796,590

## Supplementary Materials

The following are available online at http://www.mdpi.com/2073-4360/10/8/840/s1. Figure S1: The ^13^C NMR spectra of [Ch][Na] in H2O/[Ch][Na] (*R* = 10) solvent at room temperature; Figure S2: The ^13^C NMR spectra of [Ch][CH3CH2COO] in [Ch][Na] in H2O/[Ch][Na] (*R* = 10)/lignin (8 wt %) solution at room temperature; FT-IR spectra analysis of the original lignin and the regenerated lignin; Measurements of the thermal properties of [Ch][Na], [Ch][Fa], [Ch][Va] and [Ch][Sa].
