# Supplementary material for: Recyclable Choline Nicotinate and Ferulate Aqueous Solutions as Efficient Lignin Solvents
Source: Polymers (Basel). 2018 Jul 30;10(8):840. doi: 10.3390/polym10080840 (PMC6403532; doi:10.3390/polym10080840)
Supplement: Supplementary file 1 [file polymers-10-00840-s001.pdf]

Supplementary Information

Recyclable choline nicotinate and ferulate aqueous solutions as efficient lignin solvents

Airong Xu,[1,*] Lin Chen[1], Xingmin Xu[1], Zhihong Xiao[2], and Rukuan Liu[2,*]

[1] School of Chemical Engineering and Pharmaceutics, Henan University of Science and Technology, Luoyang, Henan 471003, P. R. China
[2] Hunan Academy of Forestry, Changsha, Hunan 410004, PR China

* Correspondence: E-mail: airongxu@haust.edu.cn (A.R. Xu), Tel: +86-379-64231914; liurukuan@gmail.com (R.K. Liu), Tel: +86-371-85578794

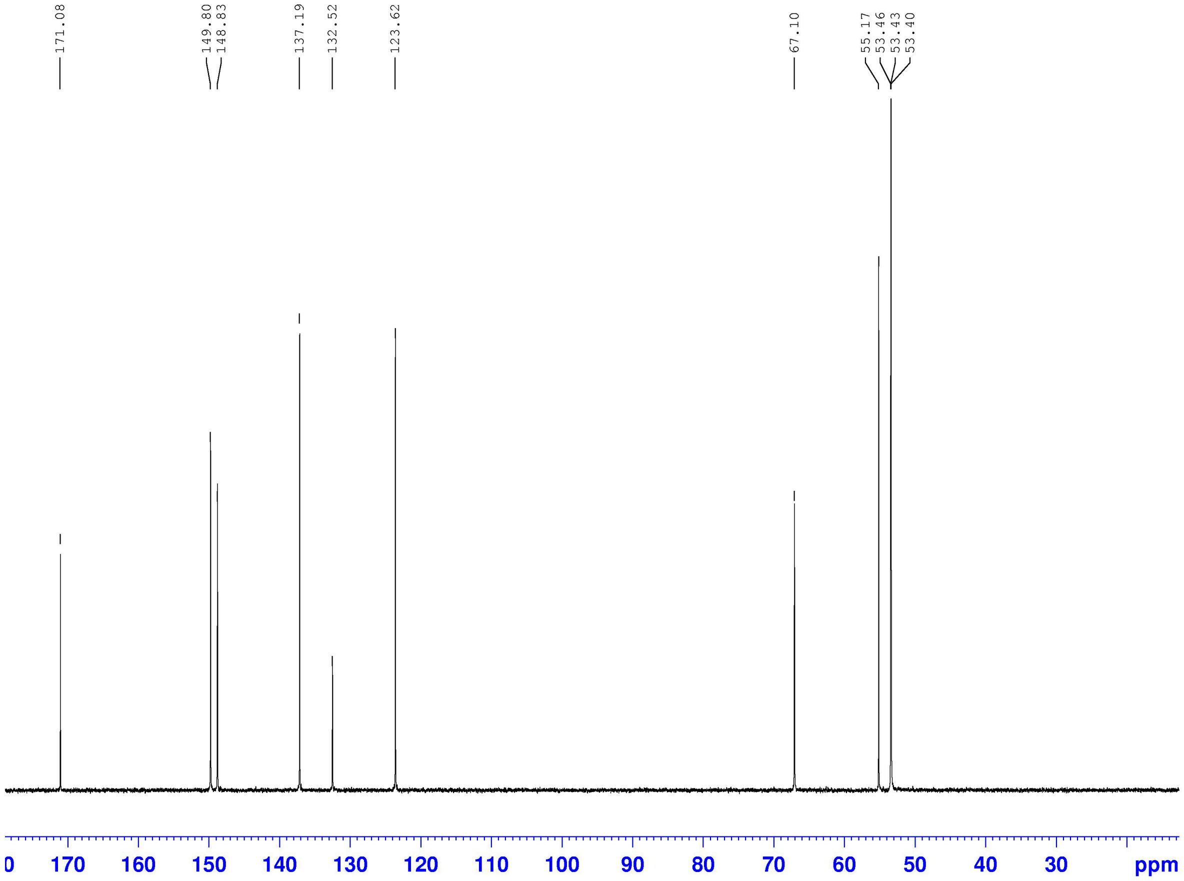

Figure S1 The ^{13}C NMR spectra of [Ch][Na] in H_2O/[Ch][Na] (R=10) solvent at room temperature

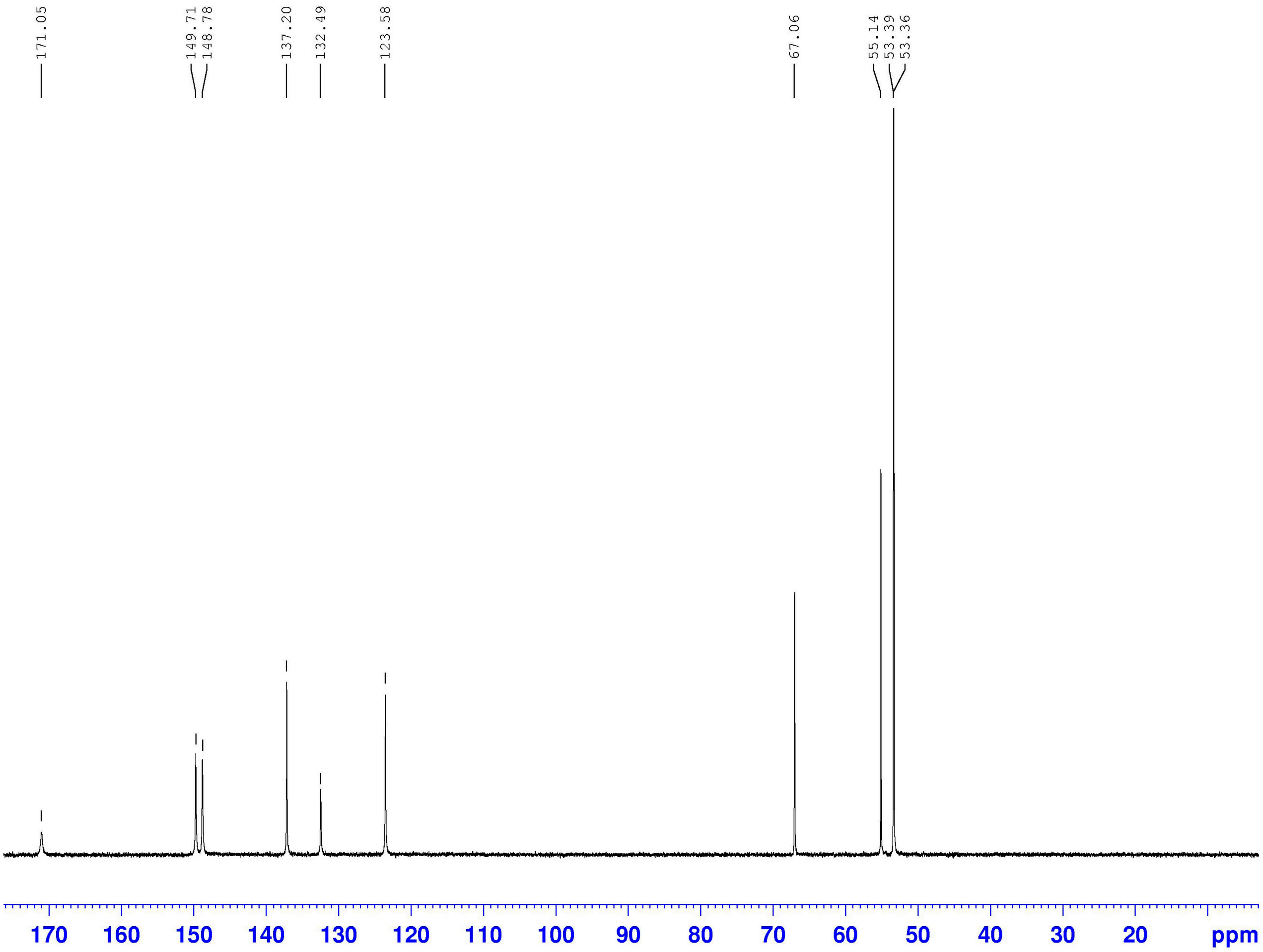

Figure S2 The ^{13}C NMR spectra of [Ch][CH_3CH_2COO] in [Ch][Na] in H_2O/[Ch][Na] (R=10)/lignin (8 wt.%) solution at room temperature

FT-IR spectra analysis of the original lignin and the regenerated lignin

The absorption band at 3429 cm^{-1} in the regenerated lignin is assigned to the stretching vibration of O-H of phenolic OH and aliphatic OH. The absorption band at 2945 cm^{-1} is assigned to the stretching vibration of C-H of CH_3 and CH_2. The absorption band at 2845 cm^{-1} is assigned to the stretching vibration of C-H of OCH_3. The absorption bands at 1600 cm^{-1}, 1515 cm^{-1} and 1425 cm^{-1} are assigned to the stretching vibration of C-C of Aromatic skeleton. The absorption band at 1460 cm^{-1} is assigned to the in-plane asymmetric deformation

vibration of C-H of CH_3 and CH_2. The absorption band at 1270 cm^{-1} is assigned to the stretching vibration of C-O of guaiacyl type. The absorption band at 1218 cm^{-1} is assigned to the stretching vibration of C–O(H) + C–O(Ar) phenolic OH + ether. The absorption band at 1136 cm^{-1} is assigned to the aromatic C-H in-plain deformation for syringyl type. The absorption band at 1030 cm^{-1} is assigned to the stretching vibration of C–O(H) + C–O(C) of 1st order aliphatic OH + ether. The absorption bands at 855 cm^{-1} and 810 cm^{-1} are assigned to the out-of-plane deformation vibration of aromatic C-H of guaiacyl type. The FTIR spectra of the original and regenerated lignin are similar to those reported in the literatures[1-3]

Measurements of the thermal properties of [Ch][Na], [Ch][Fa], [Ch][Va] and [Ch][Sa]

Melting temperature or glass transition temperature was determined on a Netzsch DSC 204 F1 differential scanning calorimetry. Each sample was sealed in aluminum pans and heated in the temperature range from -130 °C to 100 °C at a rate of 5 °C min^{-1} under dry N_2 atmosphere.

Thermal decomposition temperature was determined on a Netzsch STA 449 C thermal gravimetric analyzer (TGA). Each IL sample was heated from room temperature to 600°C in an alumina crucible with 10 wt% of mass loss at a heating rate of 10 °C min^{-1} under dry N_2 atmosphere. The temperatures reported from TGA data were the onset temperatures, which were determined from the step tangent.

References

1. Tejado, A.; Peña, C.; Labidi, J.; Echeverria, J.M.; Mondragon, I. Physico-chemical characterization of lignins from different sources for use in phenol-formaldehyde resin synthesis. *Bioresour. Technol.* **2007**, *98,* 1655-1663.
2. Xiao, B.; Sun, X.F.; Sun, R.C. Chemical, structural, and thermal characterizations of alkali-soluble lignins and hemicelluloses, and cellulose from maize stems, rye straw, and rice straw. *Polym. Degrad. Stab.* **2001**, *74,* 307-319.

3. Pinkert, A.; Goeke, D.F.; Marsh, K.N.; Pang, S.S. Extracting wood lignin without dissolving or degrading cellulose: investigations on the use of food additive-derived ionic liquids. *Green Chem*. **2011**, *13,* 3124-3136.
